# Characteristics of isolated lactic acid bacteria at low temperature and their effects on the silage quality

**DOI:** 10.1128/spectrum.03194-24

**Published:** 2025-03-17

**Authors:** La Zhu, Muqier Zhao, Yuting Yan, Pengbo Sun, Xingquan Yan, Mingjian Liu, Risu Na, Yushan Jia, Suna Cha, Gentu Ge

**Affiliations:** 1 College of Grassland Science, Inner Mongolia Agricultural University, Hohhot, China; 2 Key Laboratory of Forage Cultivation, Processing and High Efficient Utilization, Ministry of Agriculture and Rural Affairs, College of Grassland Science, Inner Mongolia Agricultural University, Hohhot, China; 3 Key Laboratory of Grassland Resources, Ministry of Education, College of Grassland Science, Inner Mongolia Agricultural University, Hohhot, China; Institute of Microbiology, Chinese Academy of Sciences, Beijing, China

**Keywords:** native grass, low temperature, silage, lactic acid bacteria, microbial community, metabolome

## Abstract

Native grasses possess rich diversity and contribute to enhancing the nutritional value of silage, promoting digestion and absorption, thus improving the health of livestock such as cattle and sheep. However, in northern China, the silage fermentation process occurs at relatively low temperatures, necessitating the use of cold-tolerant lactic acid bacteria (LAB). This study examined the effect of *Pediococcus acidilactici* (L10), a strain selected for its low-temperature tolerance, added to native grass silage at 5°C (LT), 15°C (MT), and room temperature 25°C (CK) for 60 days. The organization of the microbial community and the metabolomic profiles were examined. The results showed that temperature significantly (*P* < 0.05) influenced the pH, lactic acid (LA) concentration, and LAB populations of the silage after 60 days. The water-soluble carbohydrates (WSC) and crude protein (CP) contents in the LT treatment were significantly higher than those in the CK treatment, and the pH in the LT treatment was significantly lower than in the CK treatment. In terms of the dynamic alterations within the microbial community, *Pediococcus acidilactici* prevailed in the LT treatment, whereas *Lactobacillus plantarum* was the major genus in the MT treatment, and the CK treatment was characterized by the dominance of *Lactobacillus plantarum* and *Levilactobacillus brevis*. The study also revealed that bacterial behavior and metabolism were influenced by two-component systems and quorum sensing. At 5°C the upregulation of citric acid, salicylic acid, and L-proline was ascribed to the modification of glycolysis and the tricarboxylic acid cycle. Salicylic acid was significantly (*P* < 0.05) positively correlated with *Lactiplantibacillus plantarum*, while L-proline had significantly (*P* < 0.05) positive correlations with *Pediococcus acidilactici*, *Lactococcus lactis*, and *Weissella confusa*. These findings suggest that the addition of isolated *Pediococcus acidilactici* can enhance the quality of low-temperature native grass silage by regulating microbial metabolic pathways and community composition.

**IMPORTANCE:**

This study aimed to screen and identify low-temperature-resistant lactic acid bacteria (LAB) strains from native fermented silage of grassland pastures, evaluating their impact on silage quality in cold conditions. Under natural conditions, LAB on forage grasses are present in low numbers and exhibit insufficient activity, which is further hindered by low temperatures during ensiling, leading to slow fermentation. The findings highlighted the effects of low temperatures on the microbial community, fermentation characteristics, and metabolomic profiles of silage. After anaerobic fermentation, the main LAB strains at different temperatures were *Levilactobacillus brevis*, *Lactiplantibacillus plantarum*, and *Pediococcus acidilactici*, with *Pediococcus acidilactici* being dominant at 5°C. Temperature significantly affected the pH, lactic acid content, and water-soluble carbohydrates of silage, indicating an interaction between LAB strains and fermentation temperature. The study suggests that adding *Pediococcus acidilactici* can enhance silage quality by regulating microbial metabolic pathways and composition under low-temperature conditions.

## INTRODUCTION

Native grassland is a kind of forage resource, which is characterized by high species diversity, obvious seasonality of forage production, and medium or low nutritional value (1). Many native grasses are underutilized due to their vast diversity and seasonality. Improving animal husbandry production efficiency mainly depends on the quality, quantity, and sustainability of feed. Local native grasses feed many ruminants to meet consumer demand for meat and milk (2). In arid areas, forage resources are extremely unstable due to seasonal changes. In the rainy season, the yield of forage grass, cereals, and other forage grasses will increase, which is conducive to improving productivity. In the dry season, productivity is greatly reduced (3). The total area of meadow steppe and typical grassland in China is 400.02 million km^2^, but only 13.62 million tons of hay is supported, which is only 42% of the demand for livestock feed in winter and spring. The local herders usually start stockpiling pastures in mid-August. In the process of harvesting and storage, the dry matter (DM) and crude protein (CP) of forage decreased (4).

This has become a major factor limiting the increase in the quantity and quality of animal products in these areas (5). In this instance, other measures are required to ensure that feed supply is guaranteed throughout the year. Silage production technology is indeed a practical and economical solution. Silage is a technology that cuts, compacts, seals, and preserves green feed in a fresh state, and enables it to be preserved for long-term animal consumption through the fermentation process. Silage is easier to handle and can greatly reduce rain damage and field loss compared to hay (6). Silage is a complex biochemical process, which is determined by many factors, including temperature, moisture, nutrient composition of raw materials, harvest time, length of raw materials, packaging density, microorganisms in raw materials, and others (7). The basic principle of silage is to use lactic acid bacteria (LAB) to ferment so that it can reach a lower pH value in a short period of time while maintaining a certain anaerobic state. LAB is an important factor affecting the quality of silage (8). The key to silage is to convert soluble carbohydrates in crops into organic acids, especially lactic acid, through anaerobic fermentation. Lactic acid fermentation can better retain nutrients such as crude protein, vitamins, and minerals in crops (9). The fermentation quality of silage mainly depends on the microbial community and its metabolites. Therefore, further study on the microbial community composition of silage can provide a valuable scientific basis for improving fermentation quality (10).

In many countries around the world, especially in cold regions, the preservation of grass as silage is an important source of nutrition for livestock because it enables crops to be used throughout the year or during the limited seasonal supply of grazing animals (11). The climate of alpine pastures is harsh, the temperature is low, the frost-free period is short, and the growth season of feed crops is short, resulting in insufficient feed supply in alpine pastures (12). Environmental temperature is an important factor in determining the success of silage fermentation (13). Cao et al. reported that silage could not produce enough lactic acid to improve the quality of silage under low-temperature conditions (14). Most commercial LAB inoculants often have little or no effect on silage at low temperatures (15).

It takes a long time to harvest grasses in cold regions such as the north so that these grasses can achieve the ideal fermentation quality. Silage fermentation in cold places will also be affected by low temperatures. In general, the main fermentation type of temperate and cool silage is homogeneous fermentation (16). Some of the added silage inoculants may also be damaged as the strains are usually selected at temperatures associated with warmer climates (17). Therefore, it is necessary to screen LAB in a low-temperature environment. Screening and application of LAB strains from adverse environments can accelerate the fermentation process and better preserve the nutritional components of silage. The primary criteria of good silage candidate laboratory strains include quick growth, low pH tolerance, and rapid synthesis of metabolites such as lactic acid across a wide temperature range. Recently, some researchers have focused on the potential of low-temperature-resistant laboratory strains for silage (18). Li et al. cultivated several strains of LAB from naturally fermented silage, which showed good silage performance at a simulated temperature of 10°C–15°C on the Qinghai-Tibet Plateau (19).

In frigid climates, following a short growing season, the sudden drop in ambient temperature frequently results in partial silage fermentation or poor quality. As a result, how to properly store silage nutrients in cold climates is becoming an increasingly important issue. Farmers like to improve the quality and stability of silage by using microbial additions, however, unfavorable storage conditions can limit this benefit. Furthermore, low-temperature silage is rarely referenced in the majority of the literature on native grass silage studies. As a result, it is critical to investigate LAB that can play an active role in low-temperature circumstances, as this is a key step toward improving silage quality in northern cold regions. The features of LAB and their impact on the fermentation quality of native grasses were investigated. The study examines how different storage temperatures (5°C, 15°C, and room temperature) affect the fermentation quality, microbial population, and metabolome features of native grass silage. The goal is to provide more comprehensive and trustworthy knowledge of native grass silage additives.

## MATERIALS AND METHODS

### Lactic acid bacteria strains

Native grass in the meadow steppe of the Inner Mongolia Plateau in China was harvested at the milk stage on June 29, 2022. The following species were predominant in the meadow steppe were Chinese Leymus (*Leymus chinensis* Trin Tzvel.) and Baical Needlegrass (*Stipabaicalensis* Roshev.), Mongolian Leek (*Allium mongolicum* Regel.), Artemisia scoparia (*Artemisia scoparia* Waldst. & Kit.). According to Cai et al., 10 g samples were weighed from natural grass samples and mixed with 90 mL sterile distilled water (20). The dilutions were spread on Deman, Rugose, Sharp (MRS) agar (Difco Laboratories, Detroit, MI, USA), and incubated under anaerobic conditions at 35°C for 48 h to isolate LAB. In addition, putative homogeneous and heterogeneous LAB strains were stored in MRS containing 25% glycerol at −80°C by strain purification on MRS agar plates. The growth curve and acid production curve of the isolated LAB were determined, and a strain with fast acid production and strong growth ability was selected for the follow-up experiment. According to the study of Duan et al. (21), the production of glucose gas was detected. MRS broth was used to test the temperature resistance and sugar resistance of the strain, using API 50CH (bioMe 'rieux, Inc., Marci Itoil, France ) (20). The genomic DNA of the screened strains was screened by bacterial DNA kit (Sangon Bioengineering [Shanghai] Co., Ltd., Shanghai, China), and the isolated DNA was amplified by polymerase chain reaction (PCR) with primers 27F (5-AGT TTG ATCMTGG CTC AG-3) and 1492R (5-GGT TAC CTT GTT ACG ACT Tmur3). Then, 16S rRNA sequences were identified using BLAST analysis in the GenBank database.

### Silage preparation

Native grass at milky maturity was collected on 29 June 2023 in the Hulunbeier Meadow Steppe, Inner Mongolia, by squaring the 50 m × 100 m samples into five quadrants (100 cm × 100 cm). Using a manual feed shredder (Mode-8200; Minghong Business, Shandong, China), freshly harvested materials were cut into 2,030 mm long pieces, placed in polyethylene bags, and sealed in a vacuum. The selected LAB strain (L10) was added separately with a concentration of 105 cfu/g fresh material (FM). Fully mix FM and additives (L10: *Pediococcus acidilactici*), and pack the silage (about 250 g) in a polyethylene bag (Shenyang Huasheng Plastic Packaging Products Co., Ltd., China), then seal the bag with a vacuum sealing machine to extract air. Three temperature gradients were set in this study, room temperature (25°C) for control (CK), 15°C (MT), and 5°C (LT) for the low-temperature group. Each treatment was replicated three times, and fermentation quality was determined after 60 days of ensiling.

### Analyses of chemical composition, microorganism, and fermentation parameter

Three replicates were set up for each native grass sample. DM and content CP were measured following the method of Zhang et al. (22) and Du et al. (23). Determination of neutral detergent fiber (NDF) and acid detergent fiber (ADF) content was performed according to the method of Van Soest et al. (24) by using the Ankom A2000i fiber analyzer (Ankom Technology, Macedon, NY, USA). The water-soluble carbohydrates (WSC) content was measured following the method of Chen et al. (25). The contents of ether extract (EE) were analyzed according to Association of Official Analytical Chemists (AOAC). A sample of silage (10 g) was mixed with 90 g of deionized water following the description of Yuan et al. (26), and stored in a refrigerator at 4°C for 24 h. The leachate was filtered through four layers of gauze and filter paper, with measurements of pH, ammonia nitrogen (NH_3_-N), and organic acids in the leachate. pH was measured using a glass electrode pH meter (Leici pH S-3C, Shanghai, China). The content of LA, AA, propionic acid (PA), and butyric acid (BA) in silages was determined by high-performance liquid chromatography (HPLC; model: Waters e2695, Milford, USA) (27). The method of Broderick and Kang (28) was used to determine NH_3_-N concentrations. Microbial populations (LAB, yeasts, mold, anaerobic bacteria, and coliform bacteria) in the FM were assessed as described in a previous report (29).

### Bacterial community analysis

Native grass silage stored for 60 days was selected for bacterial community analysis. Sample DNA was extracted using the Soil Rapid DNA SPIN kit (MP Biomedicals, Solon, USA), and a Nanodrop 2000 spectrophotometer (Thermo Scientific, Wilmington, USA) was utilized for DNA concentration and purity assessment. PCR amplification was performed by Majorbio Technology Co. (Shanghai, China), and the obtained PCR products were further purified with AMPure PB beads (Pacific Biosciences, CA, United States) to prepare the SMRTbell library. The specific primers were 27F (AGR GTT TGATYNTGG CTC AG) and 1492R (TASGGHTAC CTT GTTASGACTT) primers. The purified libraries were sequenced on a Pacbio Sequel II system (Pacific Biosciences, CA, United States) using SMRT sequencing technology. The raw data were subjected to pair-end double-ended sequence splicing using Flash (version v1.2.11), and the operational taxonomic units (OTUs) were clustered using the Uparse algorithm with 97% threshold identity, and each OTU was classified using the sequence classification annotation of the RDP Classifier (version 2.13) with a confidence level of 70%. Bioinformatics analysis of plant samples was performed on the Majorbio cloud platform. Kruskal–Wallis multiple comparisons (*P* < 0.05) were used to detect bacterial community structure and analyze bacterial community structure. QIIME2 was used for α-diversity and β-diversity analysis. Community composition maps and the linear discriminant analysis effect size (LEfSe) analysis maps were plotted at https://www.omicstudio.cn/tool.

### Metabolite analysis

A 50 mg silage sample was placed in a 2 mL centrifuge tube containing grinding beads (6 mm diameter) and 400 µL of an extract mixture of methanol and water (4:1 vol ratio) containing 0.02 mg/mL of L-2-chloro chicory alanine as an internal standard was added. The samples were ground in a frozen tissue grinder at −10°C for 6 min at a frequency of 50 Hz and extracted by sonication at 5°C for 30 min at 40 kHz. After extraction, the samples were refrigerated at −20°C for 30 min, followed by centrifugation at 13,000 rpm and 4°C for 15 min. The supernatant was transferred to an injection vial containing an inserted tube to be analyzed. One QC sample was prepared for every six samples, and 20 µL of the supernatant was mixed as the QC sample. Ultra-high performance liquid chromatography-Fourier transform mass spectrometry (UPLC-FTMS) detection was performed using a Thermo Fisher UHPLC -Q Exactive HF-X analytical system with an ACQUITY UPLC HSS T3 column (100 mm × 2.1 mm i.d., 1.8 µm; Waters, USA), mobile phase A was 95% water + 5% acetonitrile (containing 0.1% formic acid) and mobile phase B was 47.5% acetonitrile + 47.5% isopropanol + 5% water (containing 0.1% formic acid). The injection volume was 3 µL, and the column temperature was set at 40°C. The samples were ionized with an electrospray source, and the mass spectrometry data were acquired in positive and negative ion modes, respectively.

### Statistical analysis

The fermentation, nutritional characteristics, and microbial counts of fresh and silage native grass were analyzed using a one-way analysis of variance (ANOVA) based on the general linear model procedure of SAS (version 9.3; SAS Institute Inc., Cary, NC, United States). One-way ANOVA and Duncan’s multiple range test were used to evaluate differences among treatments, and the effect was considered significant when *P* < 0.05. Microbiota and metabolome data were performed using an online platform of Majorbio I-Sanger Cloud Platform.3.

## RESULTS

### LAB strain characteristics

The isolated LAB strain is a homotypic fermentation bacteria (Table 1), which can not produce gas by glucose fermentation. LAB thrived at room temperature, 15°C, and 5°C, and at various glucose concentrations (5 g•L^−1^, 10 g•L^−1^, and 15 g•L^−1^).

**TABLE 1:** The selection of isolated lactic acid bacteria on the basis of the physiological tests^*a*^

Item		Strain
		L10
Gas for glucose		−
Fermentation type		Ho
Growth at temperature (°C)	5	+
	15	+
	Room temperature	+
Glucose concentration (g•L^−1^)	5	+
	10	+
	15	+

^*a*^ Ho, homo-fermentation; He, hetero-fermentation; w, weak; +, positive; −, negative.

The carbohydrate fermentation characteristics of the strain are shown in Table 2. Strain L10 could completely ferment L-Arabinose, Ribose and D-Xylose, D-Galactose, D-Glucose, D-Fructose, D -Mannose, L-Rhamnose, N-acetyl-glucosamine, Amygdalin, Arbutin, Esculin and ferric citrate, Salicin, D-Cellobiose, D-Trehalose, D-Tagatose. The results of 16S rRNA sequencing were analyzed by BLAST in the GenBank database. Strain L10 showed high similarity to Pediococcus acidilactici (100%). The nucleotide sequence of strain L10 was registered in GenBank with the accession number OP102689.1.

**TABLE 2:** The characteristics of isolated lactic acid bacteria on the base of carbohydrate fermentation^*a*^

Items	L10
L-Arabinose	+
Ribose	+
D-Xylose	+
D-Galactose	+
D-Glucose	+
D-Fructose	+
D-Mannose	+
L-Sorbitol	w
L-Rhamnose	+
Methyl-α-D-mannopyranoside	w
N-acetyl-glucosamine	+
Amygdalin	+
Arbutin	+
Esculin and ferric citrate	+
Salicin	+
D-Cellobiose	+
D-Trehalose	+
β-Gentiobiose	w
D-Tagatose	+
Potassium gluconate	w

^*a*^ W, weak; +, positive; −, negative.

### Characteristics of fresh material

The chemical composition and microbial quantity of purple native grass before silage are shown in Table 3. The DM content was 34.63%, the CP content was 16.01%, the NDF and ADF contents were 60.11% and 36.55%, respectively, the WSC content was 3.68% DM, and the fat content was 3.22%. The pH value of 6.16 was weakly acidic. The number of epiphytic lactic acid bacteria was 3.08 lg cfu/g FM, the number of yeast was 3.23 lg cfu/g FM, the number of general aerobic bacteria was 3.34 lg cfu/g FM, and the number of coliform bacteria was 3.88 lg cfu/g FM.

**TABLE 3:** Chemical composition and microbial population of native grass prior to ensiling^*a*^

Item	Content	SEM
DM/%FM	34.63	2.43
CP/%DM	16.01	0.47
WSC/%DM	3.68	0.18
NDF/%DM	60.11	2.21
ADF/%DM	36.55	0.94
EE/%DM	3.22	0.02
pH value	6.16	0.05
Lactic acid bacteria	3.08	0.04
Yeasts	3.23	0.02
Aerobic bacteria	3.34	0.05
Coliform bacteria	3.88	0.02

^*a*^ FM, fresh material; DM, dry matter; CP, crude protein; NDF, neutral detergent fiber; ADF, acid detergent fiber; WSC, water-soluble carbohydrates; LAB, lactic acid bacteria; cfu, colony forming units.

### Effects of silage temperature on nutrient composition, fermentation quality, and microbial quantity of native grass silage

Table 4 shows the chemical properties of silage fermented at different temperatures for 60 days. After ensiling, the temperature had a significant (*P* < 0.05) effect on DM content. With the decrease in temperature, the DM content of CK was significantly (*P* < 0.05) higher than that of other treatments. The ADF content of MT was significantly (*P* < 0.05) lower than that of other treatments, and there was no difference between CK and LT treatments. Similarly, as the temperature decreases, the WSC content has an upward trend. The WSC content of LT treatment was significantly (*P* < 0.05) higher than that of CK, and the WSC content of LT treatment reached 2.11%. After ensiling, the temperature had a significant (*P* < 0.05) effect on pH value and LA content (*P* < 0.05). The pH value of native grass silage treated at different temperatures ranged from 4.10 to 4.37. With the decrease in temperature, the pH value tends to decrease. The pH value of CK treatment was significantly (*P* < 0.05) higher than the other two groups. The LA content of the MT treatment was significantly (*P* < 0.05) higher than other groups, reaching 11.69%. With the decrease in temperature, the number of LAB in the MT and LT treatments was higher than that in the room temperature treatment.

**TABLE 4:** Effects of different temperatures on chemical composition, fermentation quality, and bacterial composition of native grass silage after ensiling^*a*^

Items	Treatments			*P*-value
	CK	MT	LT	
DM (%FM)	36.54 ± 0.75a	33.56 ± 0.48b	33.88 ± 0.63b	0.029
CP (%DM)	12.24 ± 0.62a	12.25 ± 0.28a	13.35 ± 0.34a	0.203
NDF (%DM)	56.93 ± 1.55a	56.71 ± 0.95a	53.13 ± 1.38a	0.152
ADF (%DM)	38.56 ± 0.01a	36.37 ± 1b	38.82 ± 0.3a	0.052
WSC (%DM)	1.79 ± 0.02b	2.03 ± 0.06ab	2.11 ± 0.13a	0.080
EE (%DM)	2.94 ± 0.36a	3.20 ± 0.07a	3.52 ± 0.37a	0.433
pH	4.37 ± 0.07a	4.12 ± 0.02b	4.10 ± 0.02b	0.007
LA (%FM)	8.82 ± 1.11b	11.69 ± 0.33a	8.81 ± 0.35b	0.042
AA (%FM)	1.59 ± 0.45a	0.81 ± 0.03a	0.48 ± 0.48a	0.187
PA (%FM)	0.91 ± 0.45a	ND	ND	0.079
BA (%FM)	ND	ND	ND	–
NH_3_-N (%TN)	0.80 ± 0.06a	0.08 ± 0.02a	0.73 ± 0.02a	0.384
LAB (lg cfu/g FM)	5.45 ± 0.40b	6.35 ± 0.10a	6.8 ± 0.09a	0.019
Yeasts (lg cfu/g FM)	5.41 ± 0.72a	6.15 ± 0.50a	6.99 ± 0.50a	0.240
Aerobic bacteria (lg cfu/g FM)	5.47 ± 0.59a	6.09 ± 0.19a	6.13 ± 0.12a	0.424
Coliform bacteria (lg cfu/g FM)	ND	ND	ND	–

^*a*^ DM, dry matter; CP, crude protein; NDF, neutral detergent fiber; ADF, acid detergent fiber; WSC, water-soluble carbohydrate; EE, ether extract; LA, lactic acid; AA, acetic acid; PA, propionic acid; BA, butyric acid; NH_3_-N, ammonia nitrogen; ND, no detected; SEM, standard error of the mean; cfu, colony-forming unit; mean values with different letters in the same row (a–b) differ significantly (*P* < 0.05). “-” indicates not detected.

There was no significant (*P* > 0.05) difference in CP content at different temperatures but the CP content was the highest in LT treatment. With the decrease in temperature, the content of NDF tends to decrease. Interestingly, the EE content also increases with decreasing temperature. The EE content of LT treatment was 3.52%. These results indicated that the silage made by adding L10 at 5°C had higher CP, WSC, EE, and lower NDF. Similarly, the content of AA decreased with the decrease in temperature. PA was not detected in MT and LT. BA was not detected under three treatments. There was no significant (*P* > 0.05) difference in NH_3_-N content among the three treatments. There was no significant (*P* > 0.05) difference in the number of yeasts and aerobic bacteria at different temperatures. Coliform bacteria was not detected at different temperatures.

### Bacterial community diversity analysis of native grass after ensiling

The bacterial community in native grass silage was analyzed using next-generation sequencing of the full-length 16S rRNA gene, as presented in Table 5. The coverage of all samples was higher than 99%, indicating that the sequencing depth was sufficient for effective bacterial community identification. There was no significant (*P* > 0.05) difference in the indexes of ACE, Chao 1, and Sobs in each treatment. There were significant (*P* < 0.05) differences in Shannon’s index between the three treatments, and the CK group was significantly (*P* < 0.05) higher than the MT and LT treatments.

**TABLE 5:** Alpha diversity of the bacterial community in nature grass silage

Items	Treatments			*P*-value
	CK	MT	LT	
Ace	60.33 ± 5.92a	48.73 ± 1.66a	45.67 ± 3.53a	0.095
Chao 1	59.44 ± 5.95a	48.33 ± 1.76a	45.67 ± 3.53a	0.116
Shannon	3.40 ± 0.l0a	2.83 ± 0.06b	3.09 ± 0.04b	0.005
Coverage	0.9999 ± 0.00a	0.9999 ± 0.00a	1.0000 ± 0.00a	0.252
Sobs	59.33 ± 5.84a	48.33 ± 1.76a	45.67 ± 3.53a	0.115

**Fig 1:**
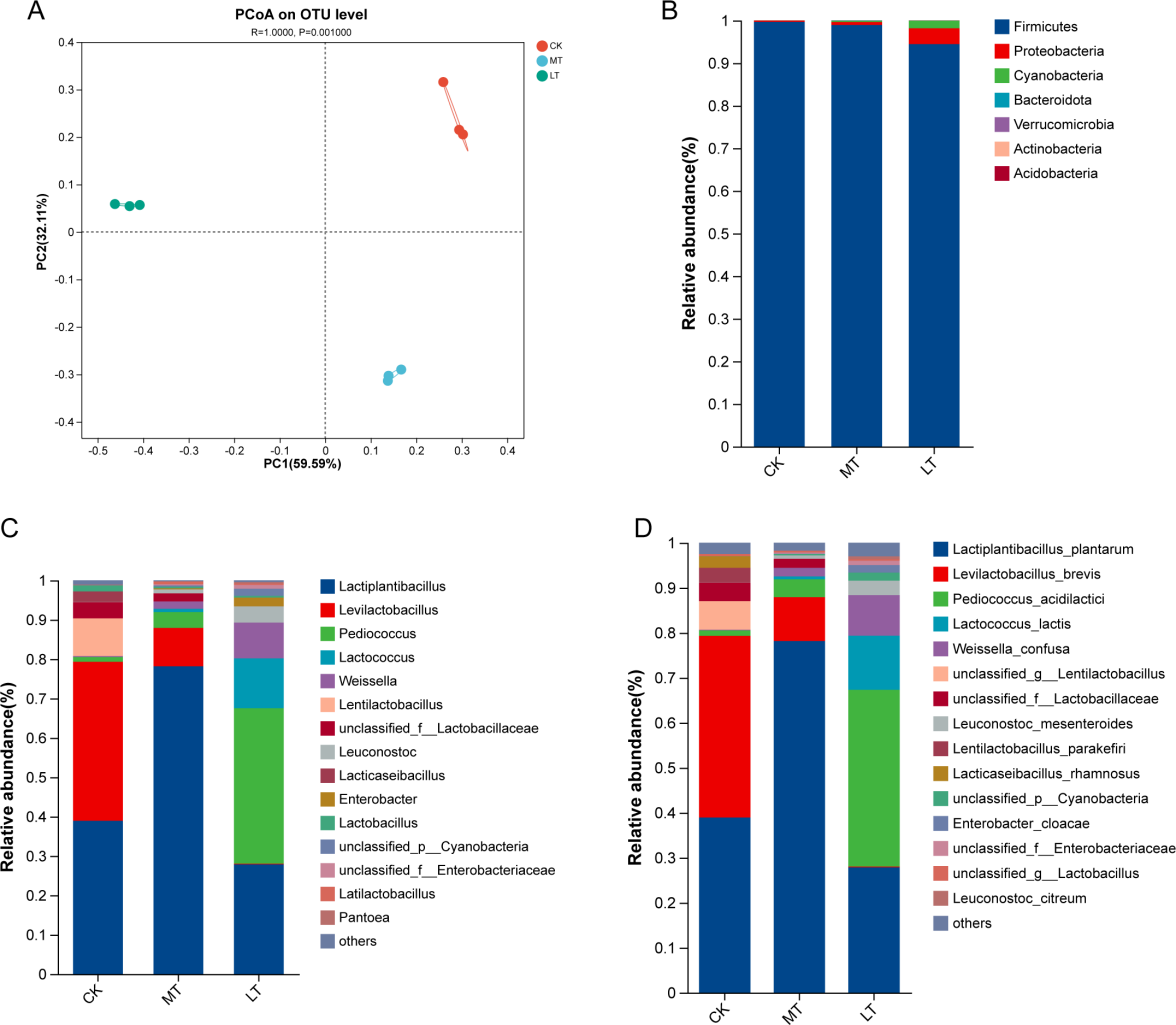
(**A**) PCoA of the bacterial community of native grass on 60 days of ensiling. Bacterial communities in 60 days of native grass silage under different treatments. (**B**) The bacterial community was shown at the phylum level. (**C**) The bacterial community was shown at the genus level. (**D**) The bacterial community was shown at the species level. CK room temperature, MT 15°C temperature, and LT 5°C temperature.

To more clearly see if the microbial community structure in the silage changed, the principal coordinate analysis (PCoA) was carried out based on the weighted UniFrac distance (Fig. 1A). Component 1 and component 2 could explain 59.59% and 32.11% of the total variance, respectively. The microbial communities at phylum, genus, and species levels in native grass silage are shown in Fig. 1B through D. At the phylum level, Firmicutes was the dominant phylum in each treatment after ensiling, and the relative proportion was more than 90.00%. At the genus level, *Levilactobacillus* of the CK group accounted for 40.40%, and *Lactiplantibacillus* accounted for 38.90%. The dominant genus of the MT treatment was *Lactiplantibacillus*, and the dominant genus of the LT treatment was *Pediococcus*, followed by *Lactiplantibacillus*. At the species level, *Levilactobacillus brevis* dominated the CK treatment accounting for 40.40%, and *Lactiplantibacillus plantarum* accounted for 38.90%. The dominant species in the MT treatment was *Lactiplantibacillus plantarum* (78.20%), and the dominant bacteria in the LT treatment was *Pediococcus acidilactici* (39.30%), followed by *Lactiplantibacillus plantarum* (27.90%). The LAB we added became the dominant bacteria at 5°C, which was what we expected.

**Fig 2:**
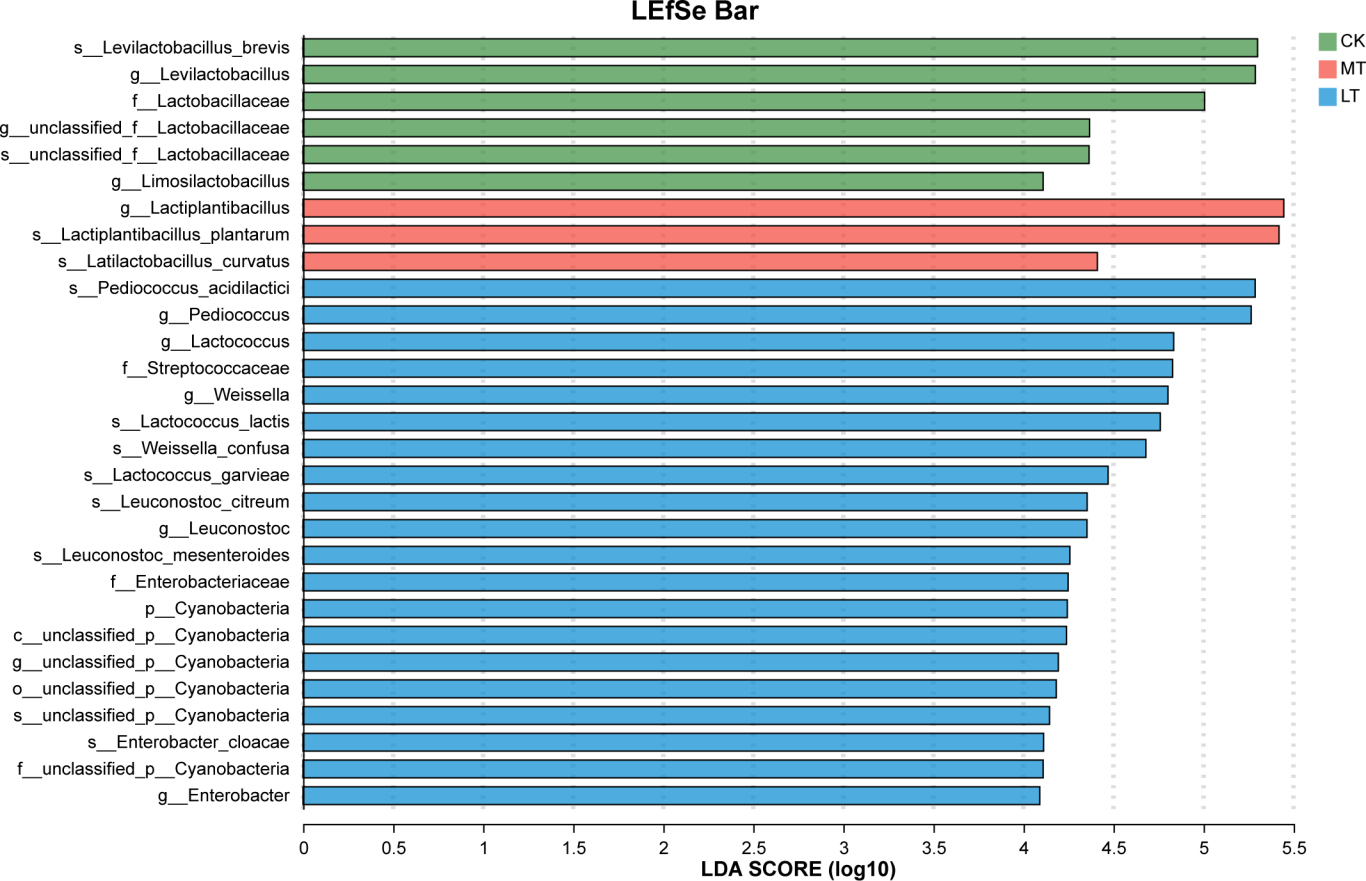
The LDA coupled on the bacterial community of native grass on 60 days of ensiling, with effect size (LEfSe) analysis. The significant difference in species was estimated by an LDA score greater at default score = 2.0. The length of the histogram shows the LDA score of differences in these groups. CK room temperature, MT 15°C temperature, and LT 5°C temperature.

The bar charts generated by LEfSe (Fig. 2) show the differences in taxa between treatments. The CK, MT, and LT treatments had a significant effect on the bacterial composition of silage at both genus and species levels (linear discrimination analysis [LDA] > 4.00). In the CK treatment, six bacteria were significantly enriched, and *Levilactobacillus brevis* showed the highest LDA score (5.30). In the MT treatment, three bacteria were significantly enriched, and *Lactiplantibacillus* showed the highest LDA score (5.45). In LT treatment, six bacteria were significantly enriched, and *Enterococcaceae* showed the highest LDA score (5.29). These results indicated that species abundance differs in specific communities at different temperatures.

### Co-occurrence networks in the bacterial community

**Fig 3:**
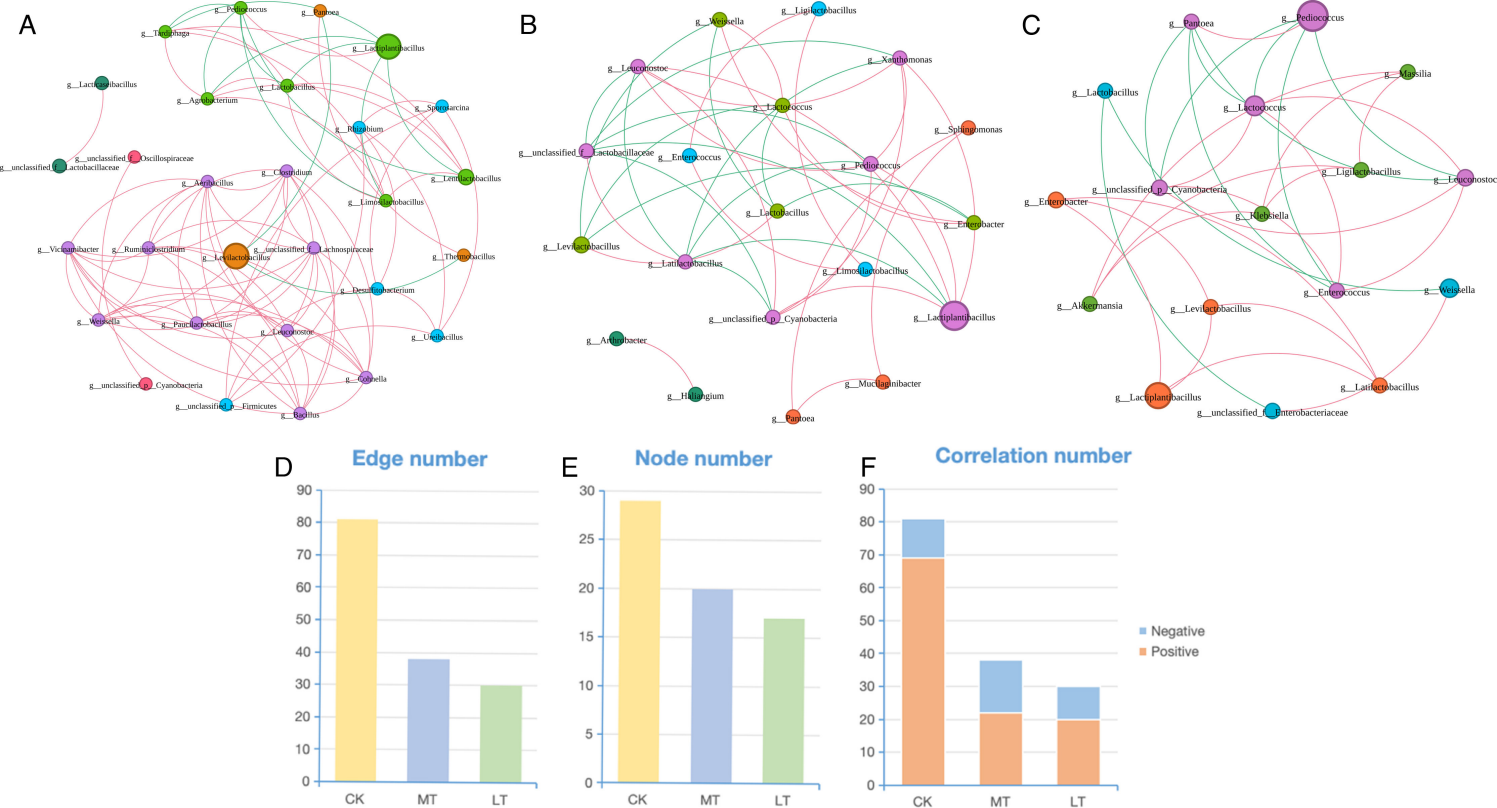
Bacterial co-occurrence networks of low-temperature native grass silage. Bacterial co-occurrence networks (Spearman correlation, the most abundant 300 species, *P* value < 0.05, correlation > 0.5) of CK (**A**), MT (**B**), and LT (C). The node represents bacterial species, node color represents bacterial genus, and node size represents the bacterial abundance. Edges are colored according to negative (green) and positive (red) correlations. (D and E) Bar plots of node and edge numbers, respectively. (F) Bar plots of negative correlation proportion and correlation number. CK room temperature, MT 15°C temperature, and LT 5°C temperature.

The bacterial co-occurrence networks of silage under different temperatures (Fig. 3A through C) were constructed to comprehensively understand the impact of storage temperature on the interactions and correlations within the resulting microbiome. The results showed that the complexity of the bacterial network was reduced at low temperatures. Compared to CK, the co-occurrence network structure of LT is simpler, as reflected in the fewer number of edges and nodes in LT (Fig. 3D through F). The greater complexity of the bacterial networks in CK in comparison to LT was in accordance with the higher (*P* < 0.05) α-bacterial diversity (Shannon) in CK.

### Differential metabolite analysis

**Fig 4:**
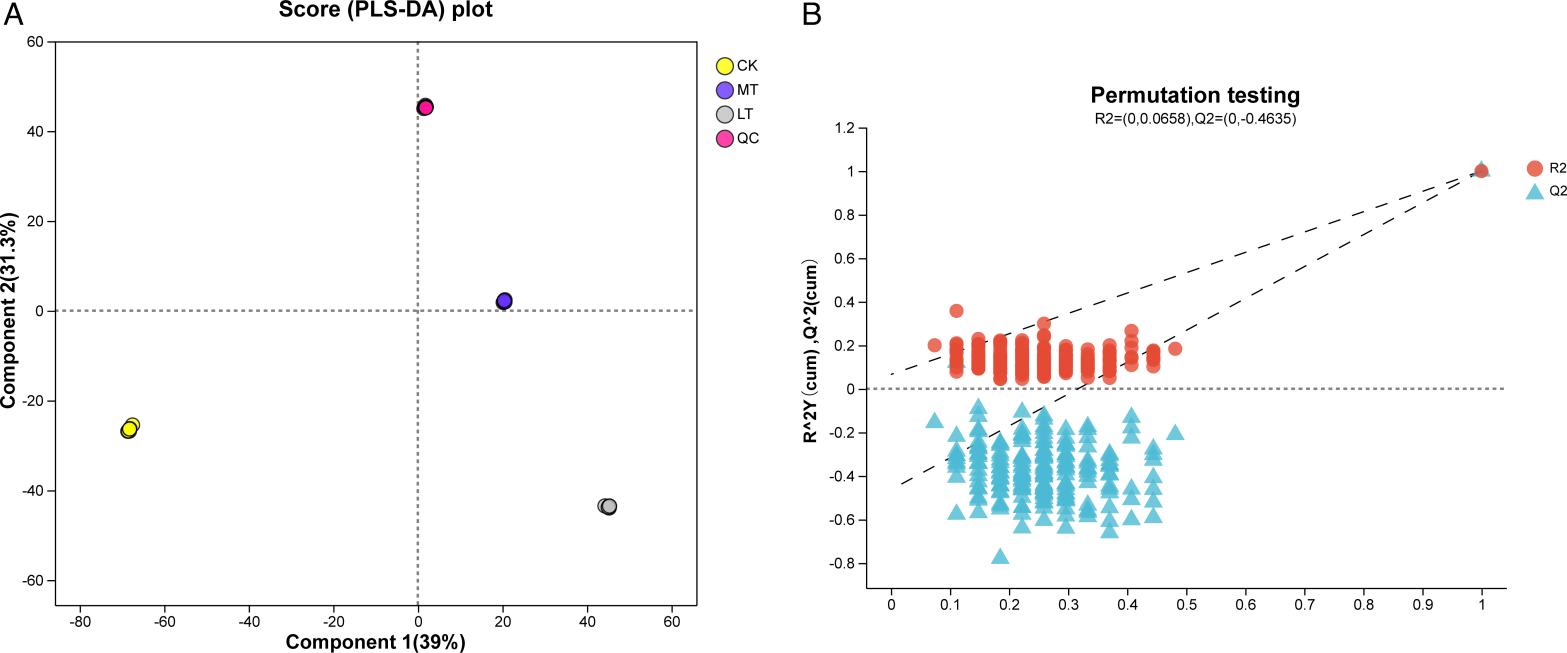
PLS-DA of the metabolic characteristics of native grass silage with the ensilage temperature. (A) PLS-DA score plot. (B) PLS-DA permutation test. CK room temperature, MT 15°C temperature, and LT 5°C temperature.

A non-targeted metabolomics approach was utilized to study the effects of isolated LAB on the metabolic products of native grass silage. Partial least squares discriminant analysis (PLS-DA) was applied to differentiate metabolites within the samples, and significant discrimination was observed among the three groups after 60 days of ensiling (Fig. 4). MT and LT treatment showed a significant ( *P* < 0.05) distance from CK treatment.

**Fig 5:**
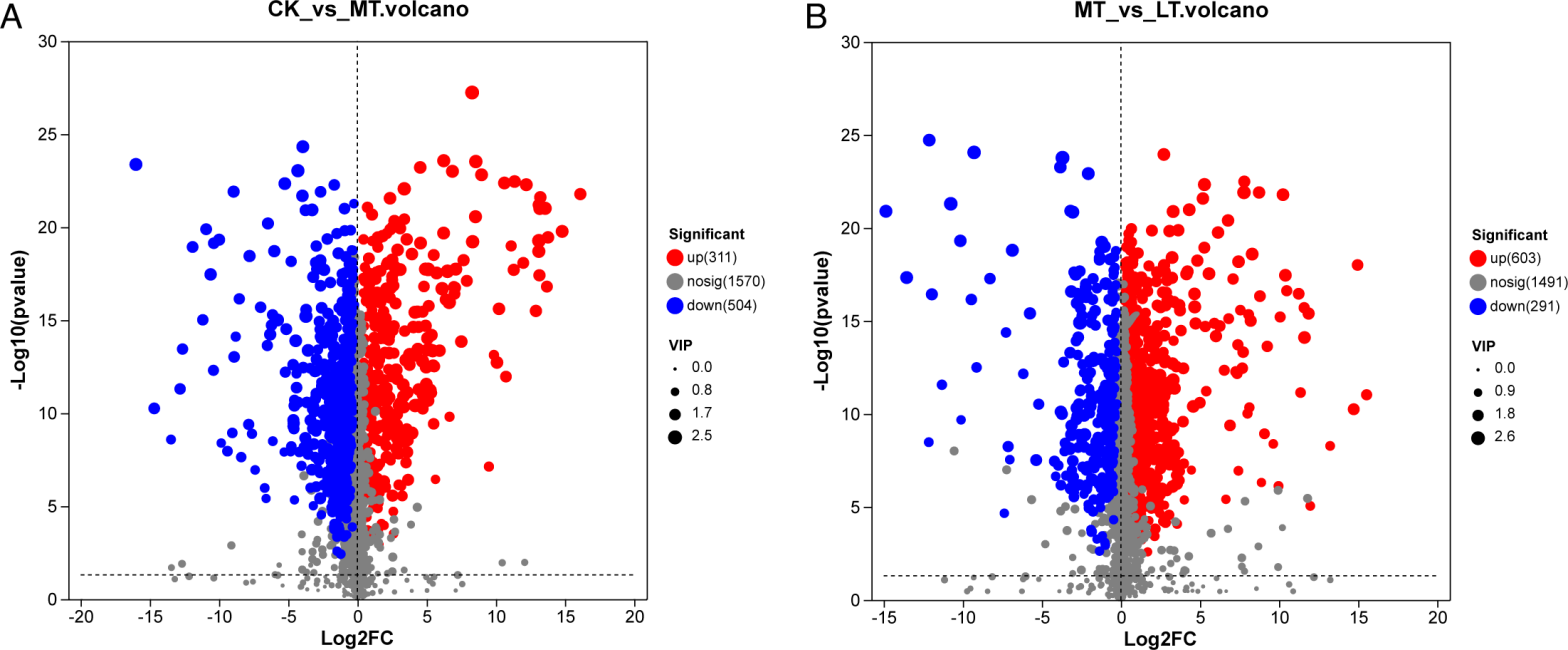
Volcano plot analysis of the differential metabolites in native grass silage with the ensilage temperature. (A) Volcano plot of CK vs MT depicts significantly upregulated and downregulated features. (B) Volcano plot of MT vs LT with similar significance classification. Log2FC on x-axis, -log10 (*P*-value) on y-axis, and VIP scores represented by dot sizes.

Differences in metabolites between CK, MT, and LT treatments were statistically analyzed by *t*-test and two-tailed test results are shown in Fig. 5. Compared with CK, 311 metabolites were up-regulated and 504 metabolites were down-regulated in the MT treatment (Fig. 5A). Compared with MT treatment, 603 metabolites were up-regulated and 291 metabolites were down-regulated in LT treatment (Fig. 5B).

**Fig 6:**
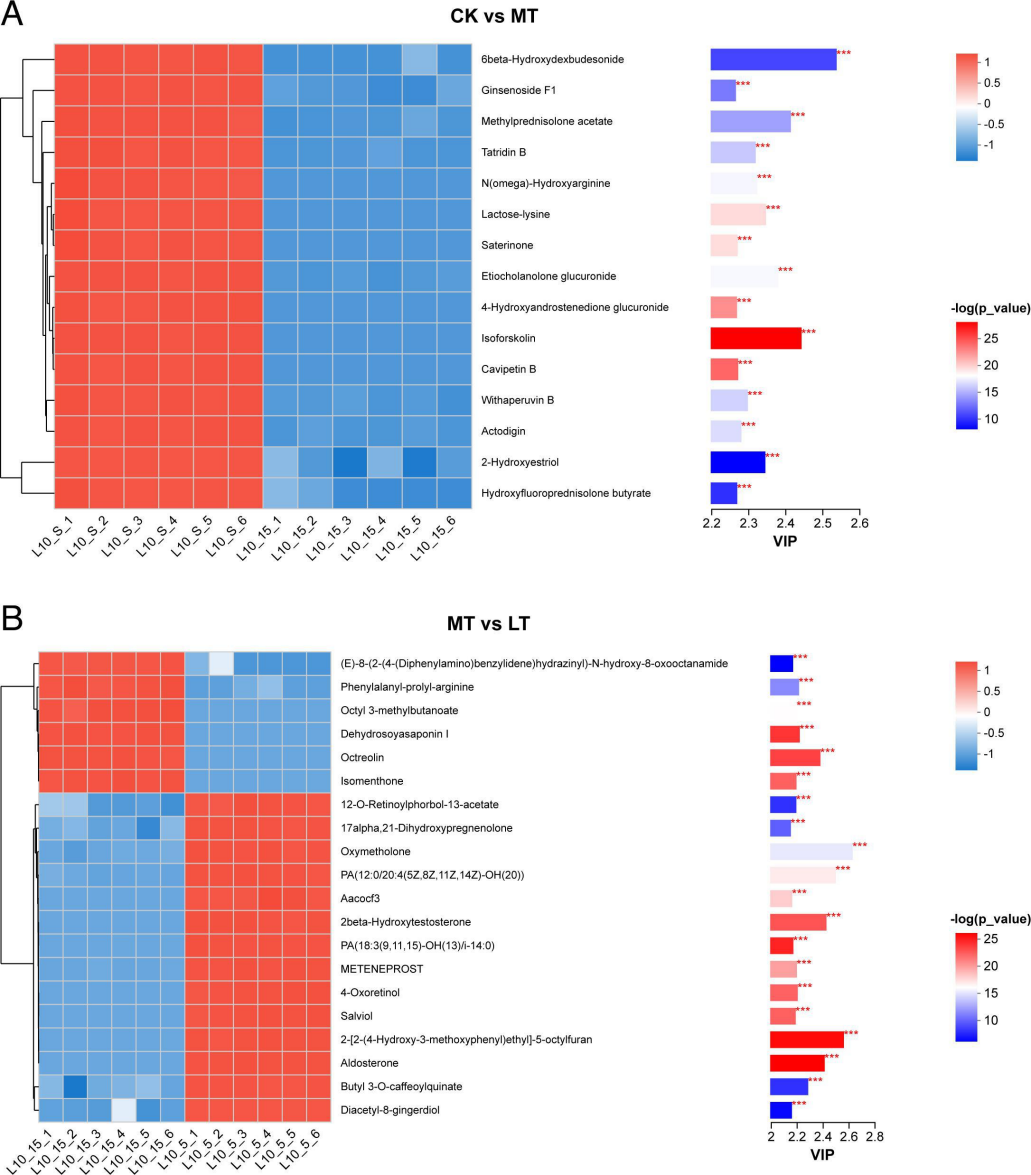
Heatmap of the differentially accumulated metabolites in native grass silage. (**A**) CK vs MT, (**B**) MT vs LT.

Based on specific screening conditions (VIP ≥ 1, *P* < 0.05), two upregulated metabolites and 18 downregulated metabolites were identified in the MT treatment when compared with the CK treatment after 60 days of ensiling. As shown in Fig. 6, 14 up-regulated metabolites and six down-regulated metabolites were identified in the LT treatment compared to MT. In the comparison between CK and MT, the up-regulated metabolites were N1,n5,n10,n14-TetraTrans-p-Coumaroylspermine and Tuliposide B, where Tuliposide B contributed greatly in this experiment. The down-regulated metabolites were 6beta-Hydroxydexbudesonide, Ginsenoside F1, Enkephalinamide-leu, Pravastatin, Desglucocheirotoxol, Methylprednisolone acetate, Tatridin B, N(omega)-Hydroxyarginine, Lactose-lysine, Saterinone, Etiocholanolone glucuronide, 4-Hydroxyandrostenedione glucuronide, Isoforskolin, Cavipetin B, Withaperuvin B, Actodigin, 2-Hydroxyestriol, Hydroxyfluoroprednisolone butyrate, where Isoforskolin contributed greatly in this experiment. In the comparison between MT and LT treatments, 2-2-(4-hydroxy-3-methoxyphenyl)ethyll-5-octylfuran contributed more to the up-regulated metabolites in this experiment. Among the down-regulated metabolites, Octreolin contributed more.

**Fig 7:**
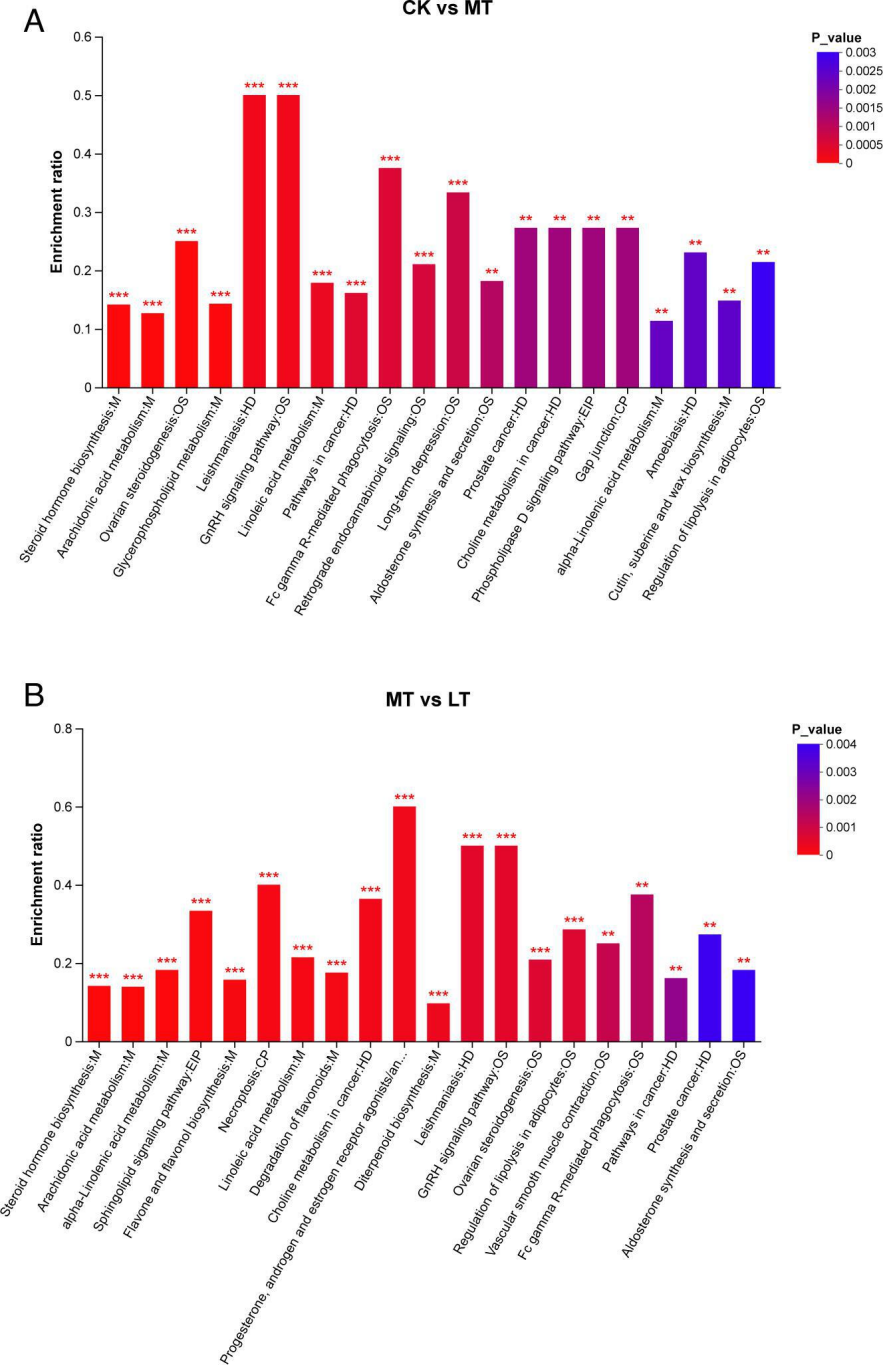
Metabolite pathway enrichment analysis following positive and negative mode ionization. Overview of metabolites enriched in native grass silage with the ensilage temperature. M, EIP, GIP, CP, OS, and HD are the class names of metabolic pathways in KEGG annotation. M, metabolism; EIP, environmental information processing; GIP, genetic information processing; CP, cellular processes; OS, organismal systems; HD, human diseases. *P*-value-corrected < 0.05 and column chart showing *P*-values for the top 20 pathways; *P < 0.05; **P < 0.01; ***P < 0.001. (A) and (B) are the heatmaps of metabolite differences of CK vs MT and MT vs LT. Colors indicate high and low levels, respectively. Right panels depict VIP scores and significance, with higher VIP values indicating greater importance in group separation.

Furthermore, enrichment analysis showed that metabolic pathways (Fig. 7). As CK treatment was compared with MT treatment, steroid hormone biosynthesis, arachidonic acid metabolism, ovarian steroidogenesis and glycerophospholipid metabolism, leishmaniasis and GnRH signaling pathways, linoleic acid metabolism, pathways in cancer, Fc γ R-mediated phagocytosis, retrograde endogenous cannabinoid signaling, and long-term depression were all significantly (*P* < 0.001) affected by different temperatures. The pathways with the highest enrichment were Leishmaniasis and the GnRH signaling pathway. Ovarian steroidogenesis and GnRH signaling are a pathway related to organismal systems, and the Leishmaniasis, pathways in cancer is a pathway related to human diseases according to the Kyoto Encyclopedia of Genes and Genomes (KEGG) Pathway database classification. In addition, such as aldosterone synthesis and secretion, prostate cancer, choline metabolism in cancer, phospholipase D signaling pathway, gap junction, alpha-linolenic acid metabolism, amoebiasis, cutin, suberine, and wax biosynthesis, regulation of lipolysis in adipocytes were significantly (*P* < 0.01) affected by different temperature, among which the phospholipase D signaling was a pathway related to environmental information processing, and the gap junction were a pathway related to cellular processes according to the KEGG Pathway database classification.

When LT treatment was compared with MT treatment, such as steroid hormone biosynthesis, arachidonic acid metabolism, alpha-Linolenic acid metabolism, sphingolipid signaling pathway, flavone and flavonol biosynthesis, necroptosis, linoleic acid metabolism, degradation of flavonoids, choline metabolism in cancer, progesterone, androgen, and estrogen receptor agonists/antagonists, diterpenoid biosynthesis, leishmaniasis, GnRH signaling pathway, and ovarian steroidogenesis, regulation of lipolysis in adipocytes were significantly (*P* < 0.001) affected by different temperature.

### Correlations between the relative abundance of bacteria and metabolites

**Fig 8:**
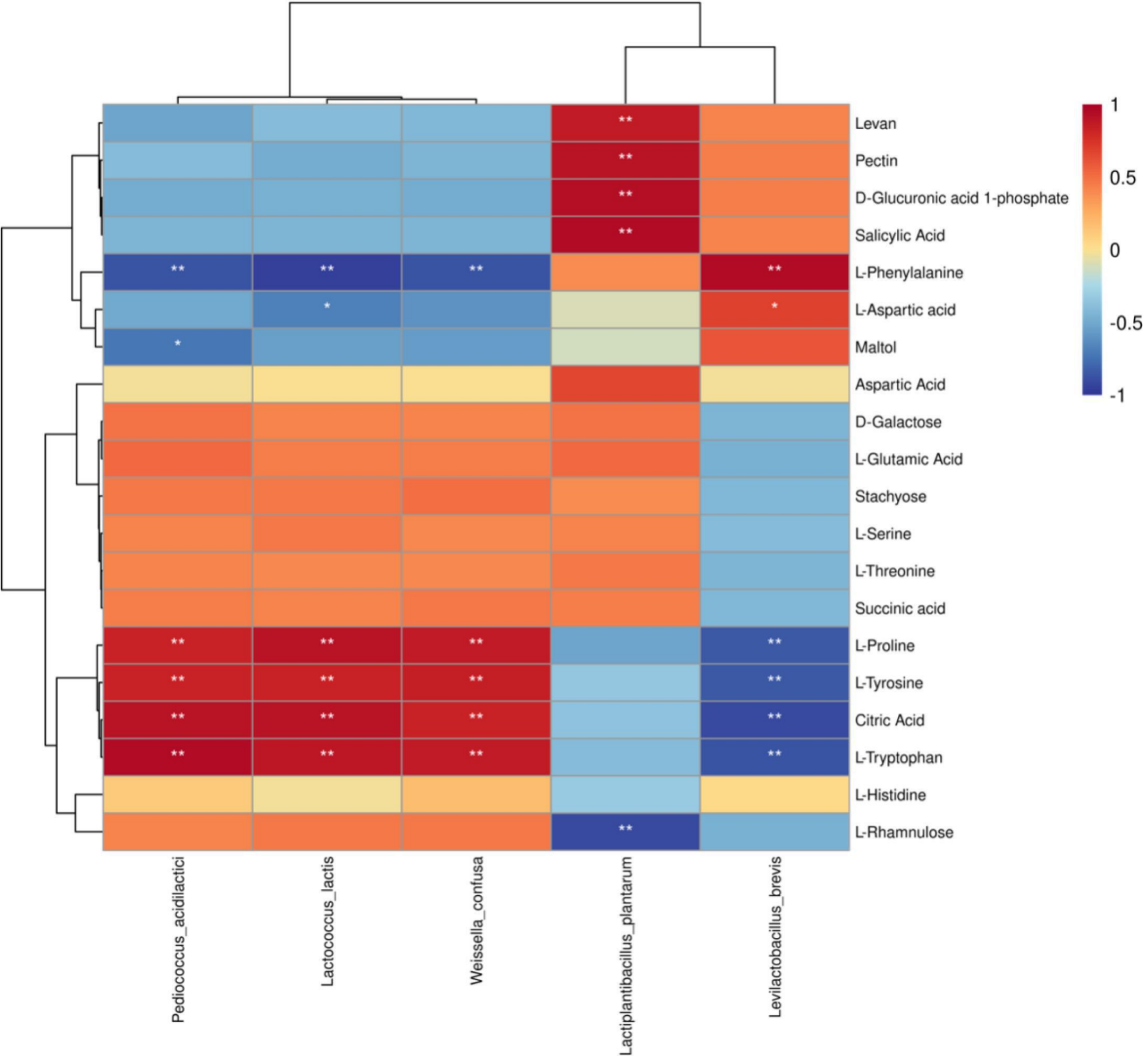
Correlation analysis of the high abundance of species-level bacteria and metabolites in silage from different temperature. *P < 0.05, **P < 0.01, respectively.

Next, we show the differential metabolites in amino acid metabolism and carbohydrate metabolism in the heat map (Fig. 8). *Pediococcus acidilactici*, *Lactococcus lactis,* and *Weissella confusa* were significantly (*P* < 0.05) positively correlated with four metabolites, including L-Proline, L-Tyrosine, Citric Acid, and L-Tryptophan, there was a significant (*P* < 0.05) negative correlation with L-Phenylalanine. The abundance of *Lactiplantibacillus plantarum* had a positive correlation with Levan and Pectin, D-Glucuronic acid 1-phosphate, Salicylic Acid but had negative correlations with L-Rhamnulose. The abundance of *Levilactobacillus brevis* had a positive correlation with L-Phenylalanine, L-Aspartic acid, and Salicylic Acid but had negative correlations with L-Proline, L-Tyrosine, Citric Acid, and L-Tryptophan.

## DISCUSSION

The use of LAB additives in silage has gained considerable attention for its critical role in improving feed quality. This study underlines the strong impact of LAB on the fermentation process and the subsequent improvement of silage quality, which supports previous studies in this field. The fermentation of silage involves a complicated interplay of LAB, environmental variables, and the intrinsic characteristics of the fodder. The successful inoculation of LAB relies on the optimization of three essential factors. Our findings underscore the necessity of choosing suitable LAB strains tailored to the forage type, alongside regulating fermentation parameters such as inoculum quantity and temperature, to attain optimal silage quality.

The use of 16S rDNA sequence analysis for identifying LAB has become a powerful and reliable tool in microbial ecology and biotechnology (30). This method, which takes advantage of both the highly conserved and variable regions within the 16S rDNA gene, allows for accurate species-level differentiation of LAB, providing valuable insights into their diversity, ecology, and functional roles. This study used 16S rDNA sequence analysis to reliably identify LAB strains. The *Pediococcus acidilactici* (L10) strain was observed to thrive at 5°C and in low glucose concentrations. This result aligns with findings from You (31), and it may be attributed to long-term evolution and native selection in the cold regions of the Inner Mongolia Plateau. Additionally, this strain can utilize a variety of carbohydrates. Due to the variation in plant raw materials, additives can impact the fermentation process during silage fermentation. To address this, we conducted a more comprehensive investigation and screening of LAB strains, considering the specific conditions of silage production. Additionally, this strain is capable of utilizing a variety of carbohydrates.

The grassland sector in China is believed to have significant development potential due to its vast grassland resources. To address the issue of forage shortages, it is crucial to make rational and efficient use of native forage. The addition of LAB proved to be an effective method for improving the fermentation characteristics of native grasses, helping to overcome their inherent limitations (32). Compared to Bao’s study (33), the DM and WSC contents of native grass in this study were relatively high. The poorer fermentation quality of the control silage may be attributed to the lower number of LAB in the raw materials (34). The content of LAB in native grass in this study is low. We employed isolated LAB as silage additions in this experiment to enhance the fermentation quality. Under low-temperature conditions, the DM content of native grass silage LT treatment was significantly lower than that of CK treatment, while the WSC content was significantly higher than that of CK treatment. Plant physiological and biochemical processes can explain this phenomenon. Under low-temperature conditions, cold treatment may also lead to changes in the lipid composition of the cell membrane, affecting the permeability and transport of solutes and thereby promoting the accumulation of soluble sugars in cells. Different patterns of metabolite accumulation occur under cold temperatures, freezing, and cold adaptation conditions. This is consistent with classical data known for decades (35). Researchers discovered that soluble carbohydrates have a key role in cold adaptation (36). Trehalose can serve as both a carbon source and an energy source in organisms at low temperatures (37). In other organisms, trehalose is a more important stress metabolite. Many biological species bodies produce and store trehalose, which directly contributes to their exceptional resilience to adverse low-temperature circumstances (38). It also provides available glycogen and trehalose for the silage fermentation process. In summary, the decrease in DM content and the increase in soluble sugar content of native grass silage under low-temperature treatment may be attributed to the adjustment of plant metabolic pathways and cell membrane composition. A pH below 4.60 during ensiling inhibits the activity of undesirable microorganisms and protein-hydrolyzing enzymes (39). Microbial metabolism converts available nutrients in WSC and DM into easily preserved organic acids and other substances (40). The NH_₃_-N content was negatively correlated with CP content. Throughout the fermentation process, both plant and microbial enzymes degrade proteins into non-protein components through proteolysis, including NH_₃_, NH_₃_-N, free amino acids, and peptides (41), which reflects the extent of protein degradation throughout the silage period (42). Additionally, the lower NH_3_-N and higher CP content in the LT treatment indicate that feed protein was well preserved after the fermentation process. This may contribute to a lower pH, inhibiting the growth and metabolism of undesirable microorganisms, such as Clostridium. Meanwhile, the digestible cell wall was broken down through microbial, enzymatic, and acid hydrolysis during the silage production process (43), resulting in a lower NDF content in the LT treatment. Maintaining a pH below 4.60 during ensiling inhibits the activity of undesirable microorganisms and protein-hydrolyzing enzymes (39). In this study, the pH values of the three treatment groups were below 4.60, a level detrimental to microbial growth. The pH of the MT and LT treatments was significantly lower than that of the CK treatment. Although low temperatures can inhibit fermentation, all silage samples obtained sufficiently low pH levels to assure anaerobic stability. The low-temperature treatment in this study had a much lower pH value than the CK treatment, which could be attributed to the addition of low-temperature-resistant *Pediococcus acidilactici*, which may have hastened the fermentation process. Previous studies have shown that adding LAB directly lowers the pH of silage, inhibits the growth of harmful microorganisms, promotes the proliferation of LAB, and increases LA content (10). Consequently, the LT treatment in this study had the highest number of LAB, although LA content was low, possibly due to the production of other acids.

High-quality silage is commonly associated with lower α-diversity (44). A higher Shannon index reflects greater microbial diversity in a sample (45) The Ace and Chao 1 indices are used to assess species richness, with lower values indicating less species diversity (46). In this study, the ACE, Chao 1, Simpson, and Shannon indices of the LP silages were lower than those of the CK silages, suggesting that inoculating low-temperature-resistant LAB at low temperatures reduced the richness (Chao 1 and ACE) and diversity (Shannon and Simpson) of the microbial community, consistent with findings reported by Li et al. (47). As Wang et al. reported, low temperature is not conducive to the growth of microorganisms other than LAB and leads to a decrease in bacterial diversity. According to PCoA analysis, the bacterial communities of the three groups were considerably segregated under different treatments, demonstrating that each group had a distinct microbiome. Therefore, we further analyzed the bacterial community. Firmicutes are the dominant bacterial phylum in silage, which aligns with the results of our study. Firmicutes were the predominant bacterial phylum in silages, consistent with the findings of our study (48). At the genus level, the three treatments were primarily composed of *Lactiplantibacillus*, *Levilactobacillus*, *Pediococcus*, *Lactococcus*, and *Weissella*, in line with previous studies (2). Zhang et al. demonstrated that alfalfa silage inoculated with *Lactobacillus plantarum* achieved the highest relative abundance of *Lactobacillus* at 30°C (49). Similarly, in this study, *Lactiplantibacillus plantarum* was dominant in the CK and MT treatments at the species level, especially in the MT treatment, where it reached 78.2%. However, a large amount of *Levilactobacillus brevis* was present in the CK treatment, with an abundance of 40.4%, even exceeding that of *Lactiplantibacillus plantarum. Levilactobacillus brevis* is a heterofermentative strain that produces both LA and AA during fermentation, thereby enhancing aerobic stability (50). The high pH value in the CK treatment may be attributed to the simultaneous dominance of *Lactiplantibacillus plantarum* and *Levilactobacillus brevis*. In the LT treatment, *Pediococcus acidilactici* had the highest abundance, followed by *Lactiplantibacillus plantarum*, *Lactococcus lactis*, and *Weissella confusa*. This suggested that at 5°C, the *Pediococcus acidilactici* added in this experiment played a leading role in fermentation. Environmental temperature influenced bacterial communities and the fermentation quality of silages, with relatively low temperatures potentially aiding in silage preservation. Environmental factors, modulation methods, fermentation period, and silage components all have a substantial impact on the microbial community networks in silage. Bai et al. (44) studied the bacterial network properties of whole-plant corn silage and found that the storage temperature had a greater influence on the network complexity than treatment with added lactic acid bacteria. Consistent with the findings of this study, the lower the temperature, the less complicated the microbial network.

In this study, liquid chromatography–mass spectrometry technology was used to analyze the metabolites of native grass silage fermentation products at low temperatures to further investigate the effect of low temperatures on these metabolites. The PLS-DA scatter plot showed significant differences in metabolites among the three sample groups, indicating that low temperatures markedly altered the metabolic composition during silage fermentation. Among the 20 most abundant metabolites, based on variable importance in projection (VIP > 2) analysis, several DEMs (including N1,n5,n10,n14-Tetra-Trans-p-Coumaroylspermine and Tuliposide B) were upregulated, and 18 metabolites were downregulated in the MT treatment compared with the control; compared with MT, LT treatment down-regulated six metabolites, including Isomenthone, Octreolin, Phenylalanyl-prolyl-arginine, and up-regulated 14 metabolites. With the decrease in temperature, MT treatment compared with CK treatment, N-Choloylglycine was up-regulated, Norepinephrine was down-regulated, Withanolide B was down-regulated; compared with MT treatment, Norepinephrine was down-regulated, Ubiquinone-2 was down-regulated, Citric Acid was up-regulated, and Withanolide B was down-regulated in LT treatment. These alterations may influence bacterial behavior and metabolism via the two-component system and the quorum sensing system, resulting in changes in the structure and composition of bacterial communities. The two-component system is one of the most important mechanisms for bacteria to detect signal molecules and regulate physiological responses, enabling them to adapt to environmental changes (51). This signal transduction system plays a crucial role in regulating cell communication and secondary metabolism. Eveliina et al. (52) found that the two-component system CheA/CheY is essential for the growth of *Yersinia pseudotuberculosis* at low temperatures. A mutation in the gene encoding the CheA histidine kinase blocks bacterial growth at low temperatures, whereas a mutation in the gene encoding the CheY regulatory protein does not affect bacterial growth under these conditions. This suggests that the two-component system is involved in the response to low-temperature stress and that the importance of its sensing and regulatory factors may vary. Liu et al. (53) found that the quorum sensing system of probiotic *Lactobacillus plantarum K25* adapts to cold stress response with a two-component system and ABC transporter, which improves the application ability of LAB in fermented food. The signal molecule AI-2 induces the response of the bacterial quorum sensing system. When the population reaches high density, it will produce a series of physiological regulation processes, including bioluminescence, movement, biofilm formation, stress resistance, metabolite accumulation, or expression of pathogenic factors (54). The bacterial quorum sensing system also influences microbial community composition. In this study, bacterial richness was higher at 5°C, and the bacterial quorum sensing system was similarly affected. Zhang et al. (55) found a strong correlation between N-acyl-homoserine lactone (AHL) content and community composition in aerobic granular sludge, indicating that AHL-mediated quorum sensing impacts bacterial community structure in this environment (56). Therefore, the composition of bacterial communities also plays a role in carbohydrate metabolism. The products of carbohydrate metabolism serve as the building blocks for many aerobic and anaerobic microorganisms, with citric acid being the main intermediate in this process (57). Therefore, the upregulation of citric acid may indicate enhanced activity of the tricarboxylic acid (TCA) cycle, which facilitates more effective oxidation of acetyl-CoA, thereby providing more energy (ATP) and reducing power (NADH and FADH2) for microorganisms. Some microorganisms may regulate the pH of the external environment by secreting organic acids (such as citric acid) to adapt to acidic conditions or improve their tolerance to acidity (58). The citric acid cycle is the final common pathway for the oxidation of carbohydrates, proteins, and lipids. TCA plays a crucial role in gluconeogenesis, transamination, deamination, and lipogenesis (59), thereby influencing silage quality. The correlation between the relative abundance of bacteria and metabolites during silage fermentation is also significant.

Salicylic acid is a compound with antibacterial activity, which may be produced during plant cell wall degradation or bacterial metabolism during silage. Its presence can inhibit the growth of certain harmful microorganisms, thereby affecting the composition of bacterial communities. At the same time, the accumulation of salicylic acid may also be linked to the high abundance of specific bacterial species. In this study, *Lactiplantibacillus plantarum* was found to be significantly positively correlated with salicylic acid, indicating that the higher the abundance of *Lactiplantibacillus plantarum*, the greater the content of salicylic acid. Anders et al. (60) reported that silage inoculated with LAB could produce salicylic acid, an antibacterial compound, which prolonged the aerobic stability of the silage. Therefore, higher salicylic acid content is associated with better silage quality. Proline is the only imino acid among the 20 standard amino acids that make up proteins. It has strong hydrophilicity and functions as a natural osmotic protectant and antioxidant. Additionally, it acts as a molecular chaperone, safeguarding protein integrity and enzyme activity (61). The study also discovered that proline has low-temperature protective properties similar to glycerol and trehalose, making it a natural, non-toxic cryoprotectant (62). In this study, L-proline, L-tyrosine, and L-tryptophan were positively correlated with *Pediococcus acidilactici*, *Lactococcus lactis*, and *Weissella confusa*. Ohshima studied the amino acid composition of alfalfa before and after silage fermentation and found that the proline in silage fermented by lactic acid bacteria was well preserved (61). Understanding the relationship between metabolites and fermentation bacteria can help improve silage quality by optimizing the fermentation process.

### Conclusion

This study demonstrated that *Pediococcus acidilactici* (L10) was successfully isolated from natural grass under low-temperature and low-sugar conditions. The isolated lactic acid bacteria were then added to natural grass silage and fermented at room temperature, 15°C, and 5°C. Our results showed that, at 5°C, the contents of WSC and CP were higher, while the BA content was lower, indicating improved fermentation quality. The dominant bacteria at 5°C was *Pediococcus acidilactici*, which was consistent with the low-temperature resistant strains we added. Through the two-component signaling pathway, bacterial quorum sensing was influenced, leading to the upregulation of citric acid and ultimately enhancing fermentation quality. The 5°C condition resulted in the most favorable outcomes in terms of microbial stability, nutrient preservation, and metabolite accumulation. Therefore, under low-temperature conditions, the addition of the isolated L10 strain to natural grass silage is recommended to improve its fermentation quality.

## Data Availability

The nucleotide sequence of strain L10 was registered in GenBank with the accession number OP102689.1. The raw sequence data were uploaded to the NCBI archive of sequence reads under study record number PRJNA1182649.
